# Expression of Concern: Benign prostatic hyperplasia wound after surgical removal in subjects on anticoagulant or antiplatelet therapy: A meta‐analysis

**DOI:** 10.1111/iwj.14303

**Published:** 2023-07-12

Ji, X, Zhao, Y, Zhang, L, Liu, Y. Benign prostatic hyperplasia wound after surgical removal in subjects on anticoagulant or antiplatelet therapy: A meta‐analysis. Int Wound J. 2022; 19(8): 1990–1999. doi:10.1111/iwj.13799. This Expression of Concern is for the above article, published online on 14 April 2022 in Wiley Online Library (wileyonlinelibrary.com), and has been published by agreement between the journal Editor‐in‐Chief and John Wiley & Sons, Ltd. The Expression of Concern has been agreed due to concerns regarding data presented in Table 2. A third party has brought to our attention that they cannot repeat this work and that some mesh terms are not accurate. As such they question the accuracy of the data in Tables 1 and 2 as they find some inconsistencies in the numbers of eligible articles based on their re‐analysis of the approach. The IWJ editorial team have been unable to reach the authors of this article for comment. The author's institution has also been contacted without success. Since we cannot verify the accuracy of the challenge or the original data, the journal is issuing this Expression of Concern to alert readers.

